# Comparison of Self-Reported and Performance-Based Emotional Granularity in Relation to Skin-Picking Behavior: An Experience Sampling Study

**DOI:** 10.3390/ejihpe15100204

**Published:** 2025-10-09

**Authors:** Albert Wabnegger, Anne Schienle

**Affiliations:** Clinical Psychology, University of Graz, 8010 Graz, Austria

**Keywords:** skin-picking, emotional granularity, experience sampling, app

## Abstract

Excessive skin-picking has been conceptualized as a maladaptive emotion regulation strategy. One potential contributor to emotion regulation difficulties is low emotional granularity (EG), defined as the ability to precisely differentiate between emotional states. To our knowledge, this is the first study to investigate whether EG for unpleasant feelings is associated with the severity of skin-picking behavior. A total of 143 individuals (mean age = 25 years, 84% female) participated in an app-assisted one-week experience-sampling study. Twice daily, they provided adjectives to describe their current affective state (performance-based EG) and rated their urge to engage in skin-picking. Additionally, they completed a Skin-Picking Scale (SPS) and an EG questionnaire (self-reported EG). Results showed that higher SPS scores were associated with lower self-reported EG (B = −0.05). However, higher performance-based EG for unpleasant feelings was linked to higher SPS scores (B = 0.02), a greater urge to engage in skin-picking (B = 0.05), and a longer duration of the behavior (B = 0.01). The two EG measures were not correlated (r = 0.01). In conclusion, these findings suggest possible biases in self-perceptions of EG in those who excessively pick their skin. Interventions that train attentional focus and promote the valuing of affective diversity may help align self-reported and performance-based EG, and in turn reduce skin-picking.

## 1. Introduction

Pathological skin-picking (PSP), characterized by excessive and repetitive manipulation of the skin resulting in lesions, psychological distress, and/or functional impairment, can be conceptualized as a maladaptive emotion regulation strategy (see the classical disorder model by Snorrason et al., 2010; and recent research by Kłosowska et al., 2024; Chesivoir et al., 2024; Schienle, 2025; Barber & Fitzgerald, 2025). Individuals with PSP often engage in skin-picking during negative affective states such as anger, anxiety, or boredom. This behavior typically serves to downregulate negative emotions, providing temporary relief (Gallinat et al., 2021; Schienle & Wabnegger, 2020; Schienle, 2025).

PSP is commonly categorized into two distinct behavioral subtypes: focused and automatic (Walther et al., 2009). Focused skin-picking is characterized by intentional efforts to engage in this behavior to eliminate perceived dermatological irregularities (e.g., bumps, scabs), typically involving visual or tactile inspection (e.g., in front of a mirror). In contrast, automatic skin-picking occurs outside of conscious awareness, often during other activities (e.g., watching television, driving), with the resultant damage (e.g., bleeding) only recognized post hoc. 

Empirical evidence suggests a strong association between PSP and difficulties in emotion regulation across both focused and automatic subtypes (see questionnaire studies by Snorrason et al., 2010; Schienle et al., 2018; Kłosowska et al., 2019; Kłosowska & Prochwicz, 2021). Snorrason et al. (2010) demonstrated that reported difficulties engaging in goal-directed behavior under emotional distress significantly predicted PSP severity. A series of studies found that individuals who infrequently utilized cognitive reappraisal—a strategy involving the reinterpretation of negative stimuli—more frequently engaged in focused skin-picking (Kłosowska et al., 2019; Kłosowska & Prochwicz, 2021). Furthermore, Schienle et al. (2018) identified a predictive relationship between impaired emotional clarity and automatic skin-picking behaviors. Kłosowska et al. (2024) expanded on this by comparing PSP individuals with a non-affected control group. The PSP group reported reduced emotional awareness.

The present study focused on a construct related to emotion regulation that has not yet been examined in the context of skin-picking behavior: emotional granularity (EG), defined as the ability to distinguish between nuanced emotional states (Barrett et al., 2001). High-EG individuals describe their emotions with precise terms (e.g., “proud”, “disappointed”), whereas low-EG individuals use vague descriptors (e.g., “good”, “bad”). Prior research shows that higher EG is associated with more effective regulation of negative affect, as individuals with high EG typically employ a broader range of strategies and are more successful in managing emotions (Gross, 2015; Kashdan et al., 2015; Kalokerinos et al., 2019). 

Individuals who engage in focused skin-picking use this behavior consciously in response to negative emotional states, which they often struggle to specifically identify (Schienle, 2025). Therefore, lower EG is likely associated with greater severity of this picking subtype, as the behavior may serve as a substitute form of emotion regulation. During automatic skin-picking, individuals exhibit reduced awareness of their emotional states, as the behavior typically takes place during attention-demanding activities. In such contexts, emotion differentiation becomes even more challenging.

Therefore, studying EG and its relation to PSP is of high clinical relevance because difficulties in differentiating emotions may undermine emotion regulation and promote maladaptive coping behaviors, such as repetitive skin-picking. Examining EG may provide new insights into underlying mechanisms of PSP and inform intervention approaches. 

In this experiment, EG was measured using both a questionnaire (self-reported EG) and app-assisted experience sampling (performance-based EG). Participants completed the Skin-Picking Scale (Mehrmann et al., 2017) and were asked to describe their current emotional state with adjectives (open-ended format) twice a day for one week. Additionally, they gave daily ratings concerning their skin-picking behavior (urge to engage in this behavior, duration of focused vs. automatic skin-picking). The objective of the study was to examine whether lower levels of EG (both self-reported and performance-based) are related to more pronounced skin-picking behavior. Specifically, we hypothesized that reduced EG would be associated with higher frequency and duration of skin-picking episodes, particularly of the automatic type (Schienle et al., 2018).

## 2. Materials and Methods

### 2.1. Participants

A total of 143 participants (120 female, 21 male, 2 diverse) with a mean age of 25.41 years (SD = 8.93) took part in this study that was advertised at social media platforms, dermatology offices, self-help groups for body-focused repetitive behaviors, and via email distribution lists at the university. Inclusion criteria were age ≥18 years and fluency in German; exclusion criteria were reported diagnoses of dermatological diseases. Seventeen percent of participants reported a current or lifetime diagnosis of a mental disorder (such as anxiety disorders, depression, or attention deficit hyperactivity disorder); however, this did not lead to their exclusion. The majority of participants had at least a high-school diploma (76%). There was no monetary compensation; psychology students were awarded course credit for their participation. 

The study followed the declaration of Helsinki and was approved by the ethics committee of the University (GZ. 39/7/63 ex 2024/25). Each participant provided written informed consent. The study was preregistered on the Open Science Framework (https://osf.io/tue3f/?view_only=be79901a7f69454eada3ff7363f2be86, accessed on 20 September 2025).

### 2.2. Research Design and Procedure

The study employed a correlational design based on experience sampling, following procedures used in previously published EG studies (e.g., Ottenstein & Lischetzke, 2020; Wabnegger et al., 2024). This design allowed us to assess performance-based EG repeatedly in daily life and to examine its association with skin-picking behaviors.

Participants first completed an online survey that assessed sociodemographic information (age, sex, and education) and included different questionnaires (see Section 2.3). Following this, participants were asked to install an app developed for momentary assessment studies (ESMira; Lewetz & Stieger, 2023) that displayed two notifications each day for a week. Notifications were randomly displayed between 08:00 am and 10:00 pm. Each notification asked participants to fill a text box by describing their current emotional state using at least one adjective (open-ended answer format). In addition, participants rated their affective state according to valence and arousal as well as their urge to engage in skin-picking on 9-point Likert scales with higher scores indicating higher pleasantness/arousal/urge (9 = maximum). Participants also estimated the duration of their skin-picking behavior in minutes per day, separately for focused and automatic picking. Participants (*n* = 23) who continued to use the app after one week (for a maximum of 11 days) were not excluded from further analyses.

### 2.3. Questionnaires and Tests

(a) The modified Skin-Picking Scale (Mehrmann et al., 2017) has nine items and measures the frequency and intensity of skin-picking as well as related impairment with a 5-point Likert scale (e.g., “How much control did you have over the urge to manipulate your skin?”). McDonald’s omega (ω) was 0.90 for the total scale. Possible sum scores range from 0–36. Scores above 7 are regarded as indicative of clinically significant skin-picking behavior (Gallinat et al., 2016).

Two additional questions (developed by the authors) referred to the degree of focused skin-picking (“I consciously engage into skin-picking behavior”) and automatic skin-picking (“I unconsciously engage in skin-picking behavior”) (0 = never; 8 = always). 

(b) The subscale Differentiation of the Range and Differentiation of Emotional Experience Scale (RDEES; Kang & Shaver, 2004) consists of seven items (e.g., “Each emotion has a very distinct and unique meaning to me”) that are rated on a 5-point scale (1 = strongly disagree, 5 = strongly agree). McDonald’s ω was 0.88 for the total scale. This scale measures self-reported EG.

(c) Two subscales of the Brief Symptom Inventory (Franke, 2000) were used that assessed the presence of symptoms of depression (6 items; McDonald’s ω = 0.77) and anxiety (6 items; McDonald’s ω = 0.77). Items are rated on a 5-point scale (0 = not at all, 4 = very strongly). 

(d) The German Multiple Choice Vocabulary Test (MWT; Lehrl et al., 1995) was used to ascertain that the sample possessed at least an average proficiency in vocabulary. The test consists of 37 items with increasing difficulty. Participants have to identify the only correct German word from a set of five words (including four non-existing words). Participants achieved a mean score of 24.55 (SD = 4.10), indicating an average verbal ability (Merz et al., 1975).

(Results for two additional scales registered in the OSF are reported in the Supplementary Materials).

### 2.4. Computation of Performance-Based EG 

To assess the specificity of the adjectives used, two experienced raters in EG studies classified each adjective as either specific (e.g., “jealous”) or unspecific (e.g., “bad”). Adjectives that did not describe an emotional state (e.g., “drunk”, “sick”) were excluded. Inter-rater reliability was good with Light’s kappa of 0.74, based on a total of 348 adjectives. Participants responded to M = 10.87 notifications (SD = 4.24). They reported a total of M = 17.50 adjectives (SD = 8.89), of which M = 5.11 (SD = 2.78) were negative.

This study focused exclusively on negative EG (including only negative adjectives), as impairments in this domain have been shown to more strongly impact emotion regulation (e.g., Barrett et al., 2001). Thus, twelve participants were excluded from further analyses because they reported only positive adjectives—leaving a final sample of 131 participants (M = 25.24 years, SD = 8.65; 85% female).

To compute a performance-based EG index, we incorporated rater agreement into the scoring procedure. For each prompt, the granularity score was computed as the product of the number of negative emotion adjectives reported and the average level of interrater agreement on their emotional specificity (i.e., 1 = specific, 0 = unspecific). For example, if the two raters classified each of three given negative adjectives as specific, the participant received a granularity score of 3. If one rater judged one adjective as unspecific while the other rated it as specific, the score was adjusted based on the average agreement. In this case, the participant received a score of 2.5 (based on the individual agreement scores of 1, 1 and 0.50). If the raters rated all three adjectives as unspecific, the participant received a score of 0. An individual’s overall granularity score was calculated by averaging these values across all completed prompts. 

As a manipulation check, we not only asked participants in the app to name their experienced feelings (adjectives) but to additionally provide ratings for the valence (pleasantness) of their affective state and experienced arousal. Negative adjectives were accompanied by lower ratings for pleasantness of the affective state (M = 4.32, SD = 1.24) than positive adjectives (M = 6.88, SD = 1.05, t(123) = −23.17, *p* < 0.001, d = −2.08). Negative adjectives were accompanied by higher arousal ratings (M = 4.27, SD = 1.62) than positive adjectives (M = 3.01, SD = 1.35; t(130) = −6.96, *p* < 0.001, d = −0.61).

### 2.5. Statistical Analysis

First, we computed intercorrelations (Pearson) between the app-based variables and questionnaire scores. Subsequently, we focused on the questionnaire scores. Possible associations between skin-picking behavior and self-reported EG (RDEES score) were assessed using hierarchical linear regression, with skin-picking severity (SPS score), anxiety, and depression (BSI) and the control variables age and sex entered at Level 1. To examine whether the degree of automatic vs. focused skin-picking explained additional variance of EG beyond Level 1, these variables were entered at Level 2 of the hierarchical regression model. 

Associations between skin-picking behavior and performance-based EG were assessed by using a linear mixed model (LMM) approach. We added the same predictor of interest (SPS score) and the following control variables (sex, age, BSI_anxiety, BSI_depression) to the model and fitted a random intercept for subjects with (i.e., performance-based granularity ~1 + sex + age + anxiety + depression + SPS + (1 I id). A second model included the same predictors as the first, with the addition of the degree of automatic vs. focused skin-picking as reported in the questionnaire survey. Model fit was evaluated using the Akaike Information Criterion (AIC), with lower values indicating better fit.

Finally, we focused on the app-based variables. A third LMM investigated the associations between performance-based EG and the reported daily urge to engage in skin-picking as well as the estimated duration of automatic and focused skin-picking per day with the control variables sex and age. 

All analyses were carried out with JAMOVI (v 2.6.44) and the GAMLj package (v 2.6.6). Results were considered as statistically significant when *p* < 0.05. Statistical assumptions (e.g., normal distributions of residuals) were tested and met sufficiently to support the validity of the analyses.

## 3. Results

### 3.1. Descriptive Statistics and Correlations

**Figure 1:**
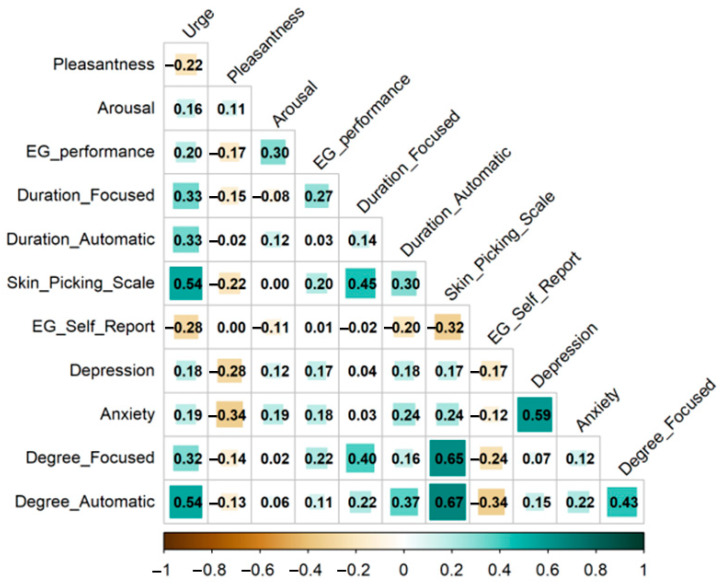
Intercorrelations between app data and questionnaires. Note: EG: emotional granularity; focused: focused skin-picking; automatic: automatic skin-picking.

Descriptive statistics for the questionnaire scores and the app ratings are shown in Table 1. Intercorrelations are depicted in Figure 1. Correlation analyses revealed significant positive associations between performance-based EG and the urge to engage in skin-picking, arousal, duration of focused skin-picking (in minutes), Skin-Picking Scale scores, anxiety, and the degree of focused skin-picking. 

In contrast, self-reported EG showed significant negative associations with Skin-Picking Scale scores, the urge to engage in skin-picking, duration of automatic skin-picking (in minutes), depression, and the degree of both automatic and focused skin-picking.

Self-reported EG was not correlated with performance-based EG (r = 0.01, [0.18, −0.16]).

### 3.2. Self-Reported Emotional Granularity 

At Level 1, results showed that, compared to males, females reported higher EG. Additionally, higher SPS scores (skin-picking severity) were associated with lower EG. For each one-point increase in the SPS score, emotional granularity decreased by 0.05 units (Table 2). The inclusion of the two skin picking subtypes (degree of automatic and focused skin-picking) did not lead to a statistically significant improvement in model fit (ΔR^2^ = 0.02, F = 1.72(2,122), *p* = 0.183).

### 3.3. Performance-Based Emotional Granularity—Predictions Based on Questionnaire Scores

SPS scores were positively associated with performance-based EG. For each one-point increase in the SPS score, EG increased by 0.02 points (Table 3). The inclusion of the two skin-picking subtypes (degree of automatic and focused skin-picking) did not result in a statistically significant improvement in model fit according to the AIC (model 1: 1552.45; model 2: 1563.74).

### 3.4. Performance-Based Emotional Granularity—Predictions Based on App-Ratings

The LMM revealed a statistically significant positive association between the urge to engage in skin-picking, the duration of focused skin-picking, and performance-based EG. Specifically, a one-point increase in the urge to engage in skin picking was associated with a 0.05-point increase in performance-based emotional granularity, and each additional minute of focused skin picking was associated with a 0.01-point EG increase (Table 4).

## 4. Discussion

This is the first study that examined the relationship between skin-picking behavior and emotional granularity (EG). EG was assessed using two complementary approaches: a performance-based measure, in which participants described their current affective states using adjectives, and a self-report measure, in which participants rated their perceived ability to differentiate between emotional states. 

Participants reported a slightly above-average level of EG on the questionnaire (M = 3.5 on a 5-point scale), while external raters evaluated EG as moderate. Interestingly, the two EG measures were not correlated with each other. This may be because performance-based EG was calculated only using negative adjectives, which were further assessed for specificity, and therefore may capture more of a state-like rather than a trait-like aspect of emotional granularity.

This finding offers a new perspective of a potential self-concept bias—that is, individuals’ beliefs about how they function emotionally may not align with their actual emotional functioning in real-time contexts. This interpretation can at least partly explain why the two EG measures showed different patterns of association with reported skin-picking behavior. Lower self-reported EG was associated with higher scores on the Skin Picking Scale (SPS, Mehrmann et al., 2017), which assesses the frequency, intensity, and consequences of skin-picking. This negative correlation aligns with our predictions, as low EG is associated with emotion regulation difficulties (Kashdan et al., 2015; Kalokerinos et al., 2019), which are thought to be a core pathological mechanism in Skin Picking Disorder (SPD). Based on the emotion regulation model of SPD by Snorrason et al. (2010), it has been suggested that individuals with SPD may rely on skin-picking as a maladaptive strategy to alleviate distressing emotional states due to limited access to more adaptive strategies, such as cognitive reappraisal (e.g., Kłosowska et al., 2019).

However, when participants were asked to describe their current emotional states via the experience sampling app, higher performance-based EG was associated with higher SPS scores, a stronger urge to engage in skin-picking and longer focused skin-picking. Thus, specific negative feelings were coupled with skin manipulation. A related finding was reported by Kłosowska et al. (2024), who also employed experience sampling in a group of participants with elevated SPS scores. In their study, a higher “Body Listening” score on the Multidimensional Assessment of Interoceptive Awareness Questionnaire (MAIA; Mehling et al., 2012) was associated with lower perceived control over skin-picking.

The present study contributes to understanding self-concept in individuals with excessive skin-picking by suggesting that they may hold distorted perceptions concerning their EG. This bias becomes particularly evident when relying on retrospective self-assessments. In a clinical interview study by Schienle (2025), patients with Skin-Picking Disorder (SPD) were asked to describe typical emotions, thoughts, and bodily sensations associated with their picking episodes. The patients reported significant difficulty in identifying (and naming) these processes. Therefore, future studies should expand the experience sampling approach to include these three dimensions. 

In addition, future studies could combine performance-based EG assessments with feedback on the specificity of adjectives used, thereby helping participants develop a more accurate perception of their EG abilities. Building on recent findings that shifts in attention to everyday affective experiences can enhance emotional granularity (Hoemann et al., 2023), experimental designs could incorporate attentional instructions alongside daily EG assessments. Such an approach would not only allow for a more precise evaluation of EG but also test whether targeted feedback and attentional training can actively improve EG and, in turn, reduce maladaptive behaviors such as skin-picking.

These approaches may be integrated into established interventions such as mindfulness-based programs or cognitive-behavioral techniques that target emotion awareness and labeling. Such combinations could offer concrete therapeutic strategies for improving EG in clinical practice.

Several limitations of the present research must be acknowledged. First, our sample consisted predominantly of females with a high level of education, which limits the generalizability of the findings to the broader population. However, it is important to note that the prevalence of skin-picking is higher among females, suggesting that our sample remains representative (Farhat et al., 2023). Second, the reported level of automatic skin-picking was not associated with EG. It should be emphasized that self-reported assessments, particularly of automatic skin-picking can serve only as proxies. Observational methods—such as video recordings and behavioral coding—may yield more reliable data. Third, although statistically significant, effect sizes were modest, and the small sample size may constrain the precision and generalizability of the findings. Thus, results should be interpreted with caution. Finally, participants reported fewer negative than positive adjectives to describe their feelings over the one-week observational period. 

In conclusion, this study provides the first evidence linking emotional granularity (EG) with skin-picking behavior, highlighting a potential discrepancy between self-perceived and actual levels of EG. Self-reported EG was negatively associated with skin-picking, while performance-based EG for negative emotions showed a positive association, suggesting a self-concept bias in individuals with excessive skin-picking. Interventions targeting EG—through feedback on adjective specificity, attentional training, or mindfulness-based programs—may help improve emotion differentiation and reduce skin-picking. Future research should refine experience sampling by providing participants with feedback on the specificity of the emotions they report.

## Figures and Tables

**Table 1:** Means, standard deviations and confidence intervals for the app ratings and questionnaires.

**Questionnaires**	**Mean (SD) [95% CI]**
RDEES-Emotional Granularity (self-reported)	3.53 (0.84) [3.38, 3.67]
BSI-Depression	0.93 (0.66) [0.82, 1.05]
BSI-Anxiety	0.95 (0.66) [0.84, 1.07]
Skin Picking Scale	9.98 (5.79) [8.98, 10.98]
Degree of focused skin-picking	4.03 (2.42) [3.61, 4.45]
Degree of automatic skin-picking	3.05 (2.17) [2.67, 3.42]
**App**	
Emotional Granularity (performance-based)	1.32 (0.61) [1.21, 1.43]
Urge to engage in skin-picking	3.59 (1.84) [3.27, 3.90]
Valence	4.26 (1.25) [4.04, 4.47]
Arousal	4.27 (1.62) [3.99, 4.55]
Duration of automatic skin-picking (minutes)	8.50 (17.42) [5.49, 11.51]
Duration of focused skin-picking (minutes)	5.75 (10.02) [4.02, 7.49]

Note: RDEES: Differentiation of the Range and Differentiation of Emotional Experience Scale; BSI: Brief Symptom Inventory.

**Table 2:** Results of the linear regression for variables predicting self-reported emotional granularity.

			95% Confidence Interval		
Predictor	B	SE	Lower	Upper	T
Intercept	3.87 ***	0.33	3.21	4.53	11.62
Age	−0.00	0.01	−0.02	0.01	−0.17
Sex:					
females–males	0.35	0.21	−0.06	0.76	1.70
diverse–males	0.01	0.61	−1.19	1.21	0.02
Depression	−0.19	0.13	−0.45	0.07	−1.41
Anxiety	0.04	0.14	−0.23	0.32	0.32
Skin Picking Scale	−0.05 ***	0.01	−0.07	−0.02	−3.79

*** *p* < 0.001.

**Table 3:** Results of the linear mixed model for questionnaire variables predicting performance-based emotional granularity.

			95% Confidence Interval		
Predictor	B	SE	Lower	Upper	T
Intercept	1.28 ***	0.16	0.97	1.59	8.17
Age	0.00	0.01	−0.01	0.02	0.68
Sex:					
females–males	−0.07	0.17	−0.40	0.25	−0.44
diverse–males	−0.41	0.46	−1.32	0.50	−0.89
Depression	0.08	0.10	−0.12	0.28	0.76
Anxiety	0.05	0.10	−0.16	0.26	0.48
Skin Picking Scale	0.02 *	0.01	0.00	0.04	2.12
**Random Effects**					
σ^2^	0.44				
τ_00 id_	0.27				
ICC	0.38				
N_id_	131				
Observations	666				
Marginal R^2^/Conditional R^2^	0.03/0.40				

* *p* < 0.05, *** *p* < 0.001.

**Table 4:** Results of the linear mixed model for variables predicting performance-based emotional granularity based on app ratings.

			95% Confidence Interval		
Predictor	B	SE	Lower	Upper	T
Intercept	1.30 ***	0.14	1.02	1.58	9.01
Age	0.00	0.01	−0.01	0.02	0.53
Sex:					
females–males	−0.11	0.16	−0.42	0.20	−0.69
diverse–males	−0.40	0.42	−1.23	0.43	−0.95
Urge to engage in skin-picking	0.05 *	0.02	0.02	0.09	2.90
Duration_Focused	0.01 ***	0.00	0.01	0.02	3.57
Duration_Automatic	−0.00	0.00	−0.01	0.00	−0.96
**Random Effects**					
σ^2^	0.46				
τ_00 id_	0.19				
ICC	0.30				
N_id_	130				
Observations	494				
Marginal R^2^/Conditional R^2^	0.06/0.34				

* *p* < 0.05, *** *p* < 0.001.

## Data Availability

Data file has been included in the Supplementary Materials.

## Supplementary Materials

The following supporting information can be downloaded at: https://www.mdpi.com/article/10.3390/ejihpe15100204/s1. Questionnaires (M, SD, confidence interval) (Izadpanah et al., 2019) and correlations with urge to engage in skin-picking (app rating) and scores on the Skin-Picking Scale (SPS) (Lischetzke, 2001).
