# Supplementary material for: Comparison of Self-Reported and Performance-Based Emotional Granularity in Relation to Skin-Picking Behavior: An Experience Sampling Study
Source: Eur J Investig Health Psychol Educ. 2025 Oct 9;15(10):204. doi: 10.3390/ejihpe15100204 (PMC12564563; doi:10.3390/ejihpe15100204)
Supplement: Supplementary file 1 [file ejihpe-15-00204-s001.zip › ejihpe-3860604-supplementary.pdf]

Supplementary Material

According to the preregistration, two additional questionnaires were completed by the participants:

a) The Heidelberg Form for Emotion Regulation Strategies (HFERST; Izadpanah et al., 2019) consists of 28 items and eight subscales: Rumination, Reappraisal, Acceptance, Problem Solving, Expressive Suppression, Experience Suppression, Avoidance, Social Support. An example item for the subscale rumination is "When I have negative feelings, I often brood over the question, why I am feeling this way." Items are rated on a 5-point scale (1 = never, 5 = always). McDonald's ω ranged between .73 (rumination) and .84 (social support) for the subscales.
b) The perception of own feelings was assessed via two subscales: emotional self-awareness (ESA) and clarity about own feelings (COF) taken from the German version of the "The perception of one's own and other's feelings" (Lischetzke et al., 2001). Both subscales consist of 6 items (e.g., I think about my feelings (ESA); "I know how I feel" (COF)) which are rated on a 4-point scale (1 = almost never, 4 = almost always). McDonald's ω was .91 (ESA) and .92 (COF).

References

Izadpanah S, Barnow S, Neubauer AB, Holl J. (2017). Development and Validation of the Heidelberg Form for Emotion Regulation Strategies (HFERST): Factor Structure, Reliability, and Validity. Assessment, 1-27.

Lischetzke T, Eid M, Wittig F, Trierweiler L. (2001). Die Wahrnehmung eigener und fremder Gefühle. Diagnostica, 47, No.4.

Correlation analyses obtained findings that are in accordance with previous research, including negative correlations between the severity of reported skin-picking behavior and the use of cognitive reappraisal (Klosowska et al., 2018) as well emotional clarity (Schienle et al., 2018). For descriptive statistics and correlations see Supplementary TableS1.

Table S1: Questionnaires (M, SD, confidence interval) and correlations with urge to engage in skin-picking (app rating) and scores on the Skin-Picking Scale (SPS)

	**M (SD) [95% CI]**	**Urge to pick**	**SPS**
Perception of own feelings			
Emotional Self Awareness	2.94 (0.66) [2.82, 3.05]	-.25**	-.38***
Emotional Clarity	3.04 (0.67) [2.92, 3.15]	-.36***	-.39***
Emotion regulation			
Acceptance	3.23 (0.85) [3.08, 3.37]	-.18*	-.19*
Rumination	3.93 (0.77) [3.80, 4.06]	.07	.12
Reappraisal	3.16 (0.84) [3.01, 3.30]	-.13	-.19*
Problem Solving	3.94 (0.65) [3.83, 4.05]	-.11	.03
Emotion suppression	3.12 (0.90) [2.96, 3.27]	-.01	.15
Expressive suppression	2.38 (0.80) [2.24, 2.52]	.04	.15
Avoidance	3.19 (0.92) [3.03, 3.35]	.25**	.15
Social support	3.60 (1.17) [3.40, 3.80]	-.07	-.18*

* $p < .05$, ** $p < .01$, *** $p < .001$
